# Supplementary material for: Implementation of reservoir computing using coupled microelectromechanical drum resonators via sideband-pumped phonon–cavity dynamics
Source: Microsyst Nanoeng. 2026 May 7;12:163. doi: 10.1038/s41378-026-01287-0 (PMC13153203; doi:10.1038/s41378-026-01287-0)
Supplement: Supplementary file 1 — Supporting information [file 41378_2026_1287_MOESM1_ESM.pdf]

Supporting information: Implementation of Reservoir Computing Using Coupled Microelectromechanical Drum Resonators via Sideband-Pumped Phonon–Cavity Dynamics

Theresa Farah[1], Loïc Flis[1], Pierre Laly[1], Guo-En Chang[2], Jun-Yu Ou[3], Yoshishige Tsuchiya[3], Yan Pennec[1], Bahram Djafari-Rouhani[1], and Xin Zhou[1,*]

[1]CNRS, University of Lille, Centrale Lille, Univ. Polytechnique Hauts-de-France, UMR 8520 IEMN, F-59000 Lille, France
[2]Department of Microelectronics, National Yang Ming Chiao Tung University, Hsinchu City 300093, Taiwan.
[3]University of Southampton, Southampton SO17 1BJ, United Kingdom
[*]xin.zhou@cnrs.fr

ABSTRACT

1 SI: Spectral density of the double-drum resonator

To measure the spectral density of the double-drum resonator, we apply a weak white noise source to artificially enhance the thermally driven Brownian motion of the mechanical modes. The applied noise has an amplitude of 10 mV, generating stochastic forces on the device. The spectral density is obtained by extending the bandwidth of the lock-in amplifier to 100 kHz. The results clearly demonstrate that the SiN drum exhibits a significantly higher signal-to-noise ratio compared to the Al drum when both resonators are driven by the same amplitude of external stochastic forces. This behavior is attributed to the significantly lower quality factor of the Al drum in the coupled system, with $\Omega_{SiN}/\Omega_{Al} \approx 2$ while $Q_{SiN}/Q_{Al} \approx 55$.

2 SI: Reservoir Computing based on the amplitude modulation of the force in the Duffing regime

Micro/nano-mechanical resonators have intrinsic nonlinear properties, coming from material properties and geometries. One well-known phenomenon is the Duffing behavior, in which the frequency response of MEMS under strong driving no longer follows the simple Lorentzian form. The motion equation of the resonator becomes :

$$\ddot{x} + \gamma_m \dot{x} + \Omega_m^2 x + \beta x^3 = \frac{F_d}{m_{eff}} \cos(\omega_d t) \tag{1}$$

where γ_m is the damping coefficient, Ω_m is the resonance frequency of the resonator, β is the Duffing nonlinearity coefficient normalized by the effective mass m_{eff} and F_d is the amplitude of the electrostatic force. For a circular membrane drum mechanical resonator (e.g. SiN drum or Al drum), the Duffing nonlinearity is primarily geometric in origin[1]. In this work, we excite only the fundamental mode of the SiN drum in the Duffing nonlinear regime. No Duffing nonlinearity was observed in the Al drum resonator.

Figure 2 (a) shows the characteristic Duffing nonlinear frequency response of the SiN drum resonator when driven by a relatively large electrostatic force. Compared with measurement results shown in the main article part, here, the resonance frequency Ω_{SiN} is lower here due to frequency drift over a measurement period of several months. For $\beta > 0$, strong driving causes the resonance frequency to shift toward higher values, and the frequency response exhibits hysteretic behavior between forward (black curve) and backward (red curve) frequency sweeps. The hysteresis window is bounded by two saddle-node bifurcation points, within which stochastic switching between bistable states can arrive[2]. For reservoir computing experiments, the operating frequency is chosen outside the bistable region at $\Omega_d/(2\pi) = 12.257$ MHz, as indicated by the dashed line in

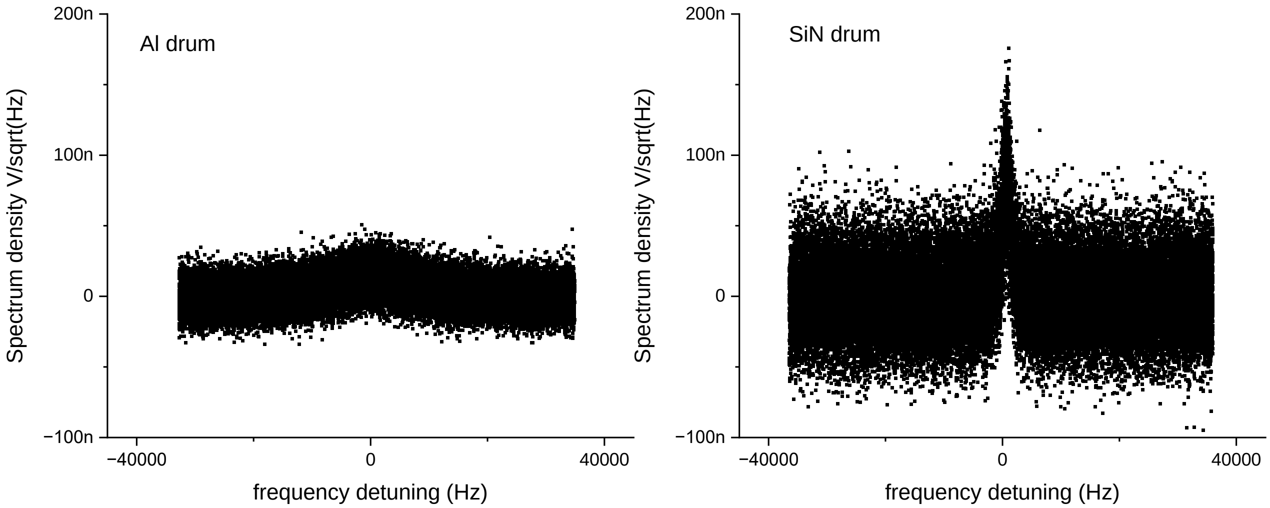

Figure 1. Spectral density of (Left) Al drum and (Righ) SiN drum resonator, measured by adding white noise with 10 mV amplitude at room temperature.

Fig. 2 (a). By fixing the drive frequency at this value, we measure the mechanical response as a function of the driving force amplitude V_d, as shown in Fig. 2 (b). Based on this response, three different driving-force modulation windows are selected for evaluating the reservoir computing performance. The $M3$ represents the whole window with variations of the drive amplitude between 0 and 16 mV where the curve shows a clear nonlinear profile. Similarly, the $M1$ showcases nonlinear variations, it is proper to drive voltage amplitudes less than 9 mV thus limiting the electrostatic drive force to smaller values. Lastly, in the $M2$ the variation of the mechanical response of the SiN membrane as a function of the drive amplitude is almost linear.

When the $M3$ modulation window is used, near-perfect success rates are achieved from the first-order (P_1) to the fourth-order (P_4) parity benchmarks, owing to the strong nonlinear variations present in this regime. As for the window $M1$, the results surpass a value of 90 % for P_1 and P_2 indicating successful predictions by our system. However, they drop to approximately 58 % starting from P_3. Moreover, for $M2$ where there is an absence of nonlinearity, the results are less important reaching 70 % for P_2 and then dropping quickly to approximately 59 % starting from $P3$.
The same conclusion can be taken, the optimal results are obtained in the modulation window that exhibits the most amount of nonlinearity in the amplitude-amplitude curve.

3 SI: Two-tone driving scheme

The coupled electromechanical resonators act as capacitor $C_g(X_1, X_2)$ consisting of 2 moving membranes. The mechanical displacement amplitudes are expressed as $X_1(t)$ and $X_2(t)$ respectively for each of the SiN and Al drum resonators resonating at frequencies Ω_1 and Ω_2. The motion equation of this double-drum resonator is described by

$$\begin{aligned} \ddot{X}_1 + \gamma_1 \dot{X}_1 + \Omega_1^2 X_1 &= \frac{V_{ac}V_{dc}}{m_1 d} C_{go} \left[1 - 2\frac{(X_2 - X_1)}{d}\right], \\ \ddot{X}_2 + \gamma_2 \dot{X}_2 + \Omega_2^2 X_2 &= \frac{V_{ac}V_{dc}}{m_2 d} C_{go} \left[-1 + 2\frac{(X_2 - X_1)}{d}\right]. \end{aligned} \tag{2}$$

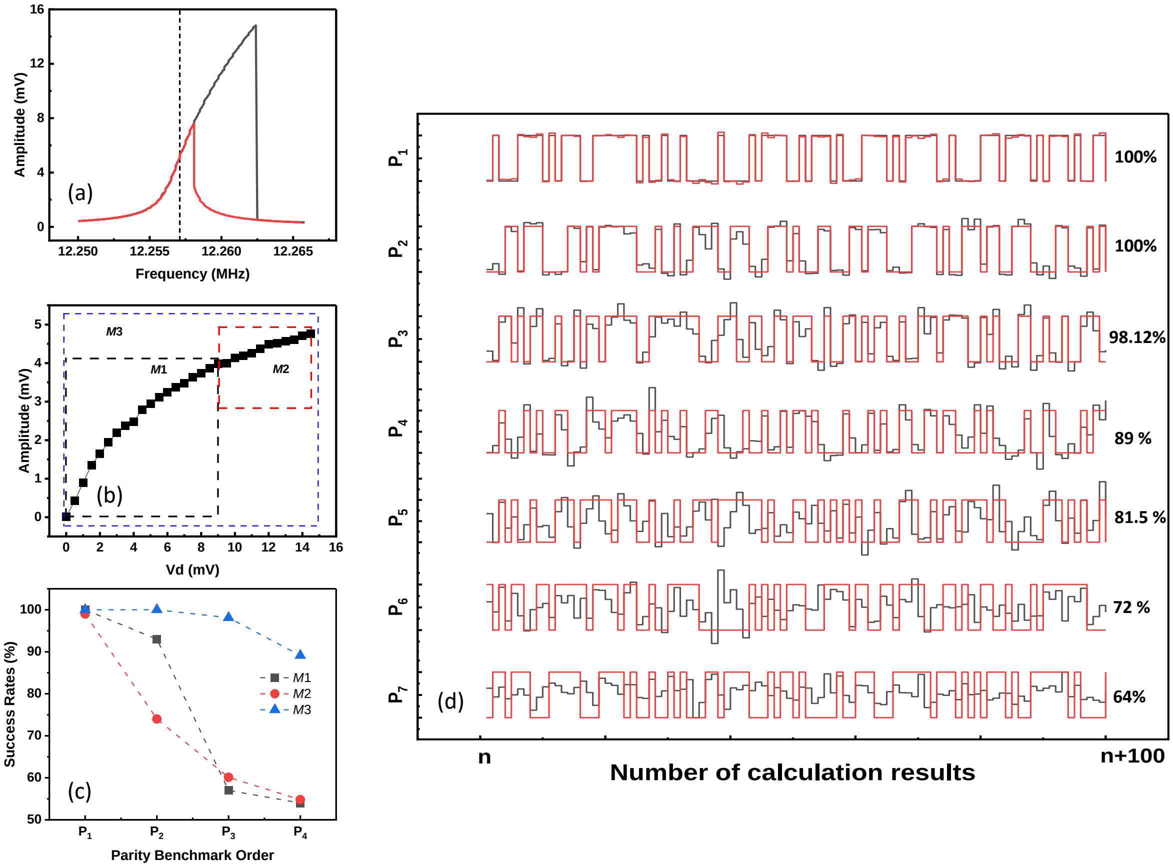

Figure 2. Reservoir Computing based on Duffing nonlinearity. (a) The mechanical response of the SiN resonator as a function of the frequency for $V_d = 15$ mV, attenuated by 20 dB. The red curve corresponds to the reverse frequency sweep and the black curve corresponds to the forward sweep. (b) The variation of the mechanical response of the resonator as a function of the drive voltage at the frequency $\Omega_d/(2\pi) = 12.257$ MHz. The curve is divided into 3 modulation windows of the electrostatic force applied: $M1$, $M2$ and $M3$. (c) The results of reservoir computing based on the modulation of the electrostatic drive force in the Duffing nonlinear regime for the 3 different modulations windows. (d) Comparison between the target output in red and the predicted output after training the output weights in black, which are obtained in the modulation window $M3$.

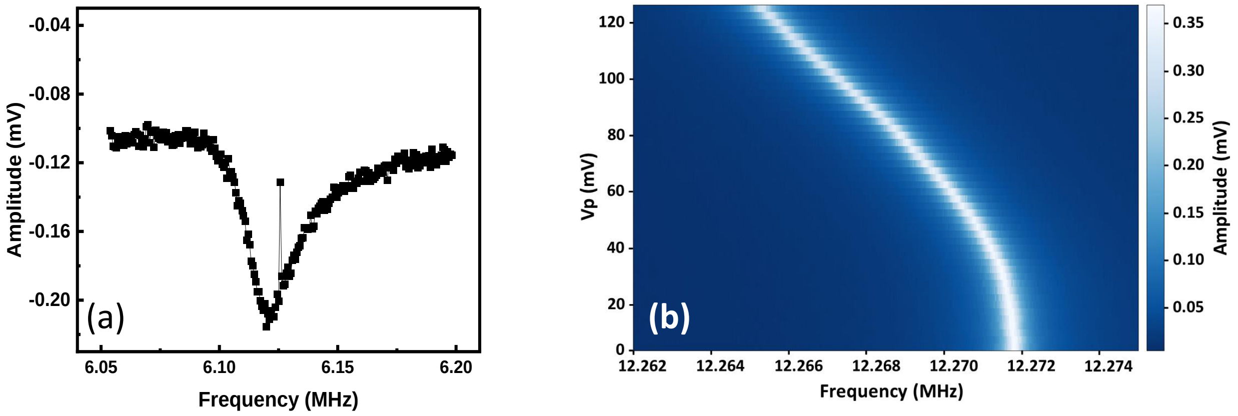

Figure 3. (a) Frequency responses of Al drum measured $V_d = 80$ mV attenuated by 20 dB, and V_{dc} = 4 V, when the phonon-cavity is pumped at its blue sideband at frequency $\Omega_p = \Omega_1 + \Omega_2 = 2\pi \times 18.3$ MHz and a pump amplitude of $V_p = 0.11$ V. (b) The 2D plots of the frequency responses of the SiN drum resonator corresponding to different blue sideband pump amplitudes.

In the blue sideband pumping scheme, $\Omega_p = \Omega_1 + \Omega_2 + \Delta$, we probe the Al drum at the frequency $\Omega_d = \Omega_2 + \delta$. The mechanical displacement x_1 and x_2 corresponding respectively to the unprobed and probed resonators are established as follows:

$$\begin{aligned} x_1 &= -\frac{f_p}{2m_1\Omega_1}\frac{x_2^*}{d}\chi_1, \\ x_2 &= -\frac{f_d}{2m_2\Omega_2}\frac{1}{\frac{1}{\chi_2} - \frac{|f_p|^2\chi_1^*}{4m_1m_2d^2\Omega_1\Omega_2}}. \end{aligned} \tag{3}$$

The $f_p = \frac{C_{g0}V_{dc}\mu_p}{d}$ and $f_d = \frac{C_{g0}V_{dc}\mu_d}{d}$ are the complex amplitudes of the pumping and the driving forces. The definition of the mechanical susceptibilities become:

$$\begin{aligned} \chi_1 &= \frac{1}{\delta - \Delta - i\frac{\gamma_1}{2}}, \\ \chi_2 &= \frac{1}{-\delta - i\frac{\gamma_2}{2}}. \end{aligned} \tag{4}$$

All derivation details have been reported in our previous work[3].

In the two-tone scheme, the interference window is primarily determined by the bandwidths of the two coupled modes, as reflected in the denominators of Eqs. 3 and 4. For the case of probing Al drum, the bandwidth of SiN γ_{SiN} is much smaller than that of Al drum, $\gamma_{SiN} \ll \gamma_{Al}$. As a result, the interference effect induced by blue-sideband pumping appears as a narrow peak within the broader bandwidth of the Al drum[3]. The pump frequency is set to Ω_{SiN} + Ω_{Al} + δ. When the swept probe frequency around the Al drum resonance reaches Ω_{Al} + δ, this interference effect becomes clearly observable, as shown in Fig.3(a).

In the sideband pumping scheme, the resonance frequency of the coupled drum resonator will shift because the pump amplitude modifies the susceptibility of the electromechanical resonator[3]. It is well-known in the optomechanical system, so called the optical spring effects. In the blue sideband pump scheme, we sweep frequency responses of the SiN drum corresponding to different pump amplitudes. As present in the Fig 3(b), the maximal resonance frequency Ω_{SiN} decreases from $2\pi \times 12.272$ MHz to $2\pi \times 12.265$ MHz when the pump amplitude increases from 0 V to 125 mV. This $2\pi \times 7$ kHz shift is still within the bandwidth of the Al membrane ($\gamma_2 \approx 2\pi \times 25.9$ kHz), however, it is much larger than that of the SiN membrane ($\gamma_1 \approx 2\pi \times 944$ Hz). In the two-tone scheme, the modulations of the pump amplitudes therefore can bring the probe tone into or out of the interference window, thereby forming a controllable nonlinear response.

References

1. Cattiaux, D., Kumar, S., Zhou, X., Fefferman, A. & Collin, E. Geometrical nonlinearity of circular plates and membranes: An alternative method. *J. Appl. Phys.* **128**, DOI: 10.1063/5.0012329 (2020).

2. Venkatachalam, S. & Zhou, X. Effects of stochastic forces on the nonlinear behaviour of a silicon nitride membrane nanoelectromechanical resonator. *Nanotechnology* **34**, 215202, DOI: 10.1088/1361-6528/acbeb0 (2023).

3. Pokharel, A., Xu, H., Venkatachalam, S., Collin, E. & Zhou, X. Coupling capacitively distinct mechanical resonators for room-temperature phonon-cavity electromechanics. *Nano Lett.* **22**, 7351–7357 (2022).
